# A clinical analysis of diabetic patients with hand ulcer in a diabetic foot centre

**DOI:** 10.1111/j.1464-5491.2010.03012.x

**Published:** 2010-07

**Authors:** C Wang, L Lv, X Wen, D Chen, S Cen, H Huang, X Li, X Ran

**Affiliations:** Diabetic Foot Care Center, Department of Endocrinology and Metabolism, West China Hospital, Sichuan UniversityChengdu, Sichuan, China; * Department of Ultrasound, West China Hospital, Sichuan UniversityChengdu, Sichuan, China; † Department of Orthopedics, West China Hospital, Sichuan UniversityChengdu, Sichuan, China

**Keywords:** diabetes mellitus, diabetic hand, hand ulcer, infection, therapy

## Abstract

**Aims:**

The aim of the study was to explore the prevalence and clinical characteristics of hand ulcer in hospitalized patients with diabetes.

**Methods:**

We analysed 17 subjects with hand ulcer among diabetic inpatients, who were admitted to the Diabetic Foot Care Center, Department of Endocrinology and Metabolism at the West China Hospital of Sichuan University from April 2003 to December 2008.

**Results:**

The prevalence of diabetic hand ulcer among hospitalized patients (0.37%) was significantly lower than that of diabetic foot ulcers (9.7%, *P*= 0.000). The mean age was 62.1 ± 9.4 years. The average known durations of diabetes and glycated haemoglobin (HbA_1c_) were 5.3 ± 4.9 years and 10.9 ± 2.4%, respectively. All patients lived in the subtropical zone. Fifteen patients (88.2%) were diagnosed with diabetic peripheral neuropathy. Ten patients had hand infection. After therapy, the ulcers healed in 13 patients (76.5%) and none of them experienced amputation. The average hospital stay for patients with local infection was characteristically longer than that for patients without infection (*P*= 0.012). The prognosis of the hand ulcer was poorer in the patients who had diabetes for > 3 years compared with those who had diabetes for < 3 years (*P*= 0.009).

**Conclusions:**

Diabetic hand ulcer is a relatively rare complication of diabetes in South-West China. Long duration of diabetes, poorly controlled blood glucose, minor trauma and delayed treatment are the risk factors. Diabetic peripheral neuropathy may play an important role in the pathogenesis of hand ulcer. Early control of blood glucose with insulin and early anti-microbial therapy with appropriate antibiotics are crucial. Debridement and drainage are necessary for hand abscesses.

## Introduction

Because of its high amputation and mortality rate, diabetic foot is receiving more and more attention as one of the severe diabetes-related complications. However, diabetic hand is less well recognized and usually overlooked by clinicians. In fact, diabetic hand has, to date, not been considered as a specific complication of diabetes and no precise definition for it can be found in the literature. Papanas and Maltezos [1] suggested that it could be defined as a syndrome of musculoskeletal manifestations of the hand in diabetic patients. Although limited joint mobility, Dupuytren’s contraction and trigger fingers are the most commonly published conditions of the diabetic hand, hand ulcer with infection can cause more serious complications such as gangrene [2], amputation [3,4] or even death [5]. Hand ulcer with infection in diabetic patients was first described in the USA in 1977 [4]. However, since 1983, the subsequent majority of reports [6] were from African countries, and hand ulcer with infection was also termed the tropical diabetic hand syndrome [2]. Excluding a few case reports, there has been no analysis of the clinical characteristics of hand ulcer in Chinese diabetic patients. To understand the clinical features and risk factors of hand ulcers in hospitalized patients with diabetes, we studied diabetic inpatients with hand ulcer in a diabetic foot care centre over a period of 5.75 years.

## Patients and methods

We reviewed the medical records of the diabetic inpatients admitted to the Diabetic Foot Care Center, Department of Endocrinology and Metabolism at the West China Hospital from 1 April 2003 to 31 December 2008. Inclusion in this study required patients with at least one ulcer in the hand. We examined the discharge diagnoses of hand ulcer or diabetic hand. Detailed hospital records of these patients were retrieved and available information, including clinical history, laboratory profiles, microbiological analysis, therapeutic course and outcome, were collected. A poor prognosis indicated that the ulcer did not heal or the patient died within 1 year after discharge.

All calculations were performed using SPSS version 13.5 software (SPSS Inc., Chicago, IL, USA). The data were expressed as means ± sd when variables were normally distributed. Frequencies and percentages were used for statistical descriptions. Continuous variables were analysed using Student’s *t*-test. Univariate testing was performed using the χ^2^-test for categorical variables. Statistical significance was defined as *P*< 0.05.

## Results

### The prevalence of hand ulcer in diabetic inpatients

During the period under review, 4615 diabetic patients were admitted to the Diabetic Foot Care Center, Department of Endocrinology and Metabolism. Of those inpatients, 17 had hand ulcers, one with Type 1 diabetes and 16 with Type 2 diabetes. The prevalence of diabetic hand ulcer was 0.37%, significantly lower than that of diabetic foot ulcers (9.7%, *P*= 0.000).

Of the 17 patients [mean age 62.1 ± 9.4 years (range 43–79 years)], 13 were men with a long smoking history and four were women. Their body mass index (BMI) ranged from 18.6 to 28 kg/m^2^, mean 23.1 ± 3.2 kg/m^2^. The average known durations of diabetes was 5.3 ± 4.9 years, and mean HbA1c 10.9 ± 2.4%. Only two had access to higher education, none had a diabetic family history. Twelve patients took oral anti-hyperglycemic drugs and two used insulin before hospitalization. Three had newly diagnosed Type 2 diabetes.

Paraesthesia and tactile hypoesthesia were present in 15 patients (88.2%). An electromyogram was performed in four, showing a deficit of sensory and motor nerve conduction velocities in the upper and lower extremities. A vascular colour Doppler ultrasound (Philips iU22 ultrasound system, Philips Co. Ltd, WA, USA) revealed atherosclerotic plaques of lower limb arteries in 14 patients (82.3%). Embolism of the left ulnar artery was shown in one patient, and multiple stenoses and occlusion of arteries in upper and lower limbs were present in one patient with end-stage renal disease.

Retinopathy was diagnosed in five subjects (29.4%) and nephropathy in eight (47.1%), including five patients with microalbuminuria, two with clinical proteinuria and one with end stage renal disease. Five patients (29.4%) had diabetic foot ulcers.

### Mode of presentation of hand lesions

**FIGURE 1:**
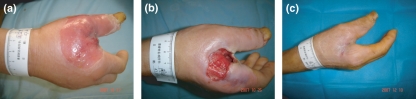
Abscess of the right hand in a diabetic patient: (a) the third day after admission; (b) 1 week after debridement and drainage; (c) hand ulcer healed in the sixth week after autologous platelet-rich gel therapy.

Twenty-nine ulcers in hands were identified, including dry gangrene in two patients, abscess (Fig. 1a) in seven, osteomyelitis in nine, and superficial ulcers in eight. Ten patients (58.8%) had evidence of concomitant infection in 16 ulcers. Bacterial and fungal cultures were performed in only 12 patients, and the organisms isolated were *Staphylococcus aureus* (*n* = 3; 25%), streptococcus group D (*n* = 1), colon bacillus (*n* = 1), corynebacterium (*n* = 1) and fungus (*n* = 2). One patient had a mixed infection with *Staphylococcus aureus* and fungus. No organism was cultured from the ulcers in five patients.

### Location and causes of the hand ulcers

The left hand was involved in nine patients and the right hand in 10 subjects. Two patients had bilateral hand involvement. Twenty-one ulcers were located on the fingers (16 ulcers were on the third, fourth and fifth fingers), two ulcers were on the dorsal surface of the hand and six were on the palms of the hands.

Only seven patients could describe the possible causes of the ulcers, which included stab wounds, injury caused by clipping nails, local eczema, chilblain and scald.

### Treatment and outcomes

All of the patients received intensive insulin therapy, intravenous antibiotics and dressing changes. Debridement was performed on the infected ulcers in 10 patients. Seven patients underwent incision and drainage of an abscess. Elevation and immobilization of the hands with infection were required. Autologous platelet-rich gel was used in two patients to improve ulcer healing (for example, see Fig. 1b and c).

The hand ulcers healed in 10 patients (58.8%) during hospitalization. The ulcer size of five patients decreased obviously; of those, three subjects required outpatient wound care and healed within 1 month after discharge, one died of respiratory failure within 1 week after discharge and the patient with dry gangrene died 1 year later as a result of uraemia. Two patients discharged themselves against medical advice because of economic difficulties, one of whom now still has dry gangrene in his right hand and another one reported missing in the earthquake in 2008.

The mean hospital stay for all patients was 38.2 ± 27.5 days (range 7–85 days). The average length of hospital stay for those who had a local infection (48.1 ± 26.4 days) was significantly longer than those without infection (25.4 ± 24.9 days, *P*= 0.012). The prognosis of the hand ulcer was considerably poorer in the long-term diabetic patients (known duration of diabetes > 3 years) compared with the short-term diabetic patients (known duration of diabetes < 3 years) (*P*= 0.009).

## Discussion

The prevalence of diabetic hand ulcers (0.37%) in our Diabetic Foot Care Center was significantly lower than that of diabetic foot ulcers (9.7%). The prevalence rate of diabetic hand [6–8] has been reported to be 1.4–3.2% in African countries. This difference may have resulted from the difference in geographic locations. Sichuan province, located in South-West China, belongs to a subtropical zone with a monsoon climate. Most African countries are situated in the tropical area, where insect bites and stings are very common. A neglected minor wound is more likely to be infected by bacteria in a hot and humid environment. Compared with a predominance of women with diabetic hand ulcers in Africa [4,8], in our study, hand ulcers were more common in men. The explanation may be that males are the main labour force of a family in China, while females are responsible for manual labour in Africa.

A relatively long duration of diabetes (mean 5.3 ± 4.9 years), poorly controlled blood glucose (mean HbA_1c_ 10.9 ± 2.4%) in elderly patients (mean 62.1 ± 9.4 years) and delayed presentation to the hospital because of poor educational background and lack of knowledge about diabetes might contribute to the occurrence and deterioration of hand ulcer in our study, a finding similar to another report [9]. The *Staphylococcus aureus* infection rate in this study was lower than that in a previous report (44%) [10], but this was still the main pathogen in the hand ulcers.

Diabetic peripheral neuropathy was present in 88.2% patients, but ischaemia of the upper extremities only existed in two subjects. Abbas and Archibald [11] showed that peripheral neuropathy was predominant in patients with the tropical diabetic hand syndrome compared with their control group. We suggest that unawareness of impaired sensation in hands with insidious trauma could be associated with diabetic hand ulcers in South-West China, and long-term hyperglycaemia could aggravate the infection of a hand wound.

In total, the ulcers in 13 (76.5%) of our patients healed, and none of them experienced amputation. However the tropical diabetic hand syndrome can lead to permanent disability and limb amputation; Abbas and Archibald reported that 13% of their patients required major limb amputation, or even to death [11]. Gill *et al*. [9] suggested that prompt amputation if necessary was one of the management strategies for tropical diabetic hand ulcers. Compared with African patients, hand ulcers in Chinese diabetic patients became worse more slowly, with a comparatively better prognosis.

In conclusion, our study demonstrates that diabetic hand ulceration is a relatively rare complication of diabetes in South-West China. Long duration of disease, poor metabolic control, minor trauma and delayed presentation are the risk factors, and diabetic peripheral neuropathy may play an important role in the pathogenesis. Early control of blood glucose with insulin and early anti-microbial therapy with appropriate antibiotics are important. Debridement and drainage are necessary for an abscess in the hand.

## Abbreviation

HbA_1c_: glycated haemoglobin
